# Cardiovascular magnetic resonance (CMR) in restrictive cardiomyopathies

**DOI:** 10.1007/s11547-020-01287-8

**Published:** 2020-09-24

**Authors:** Nicola Galea, Gesualdo Polizzi, Marco Gatti, Giulia Cundari, Michele Figuera, Riccardo Faletti

**Affiliations:** 1 Department of Experimental Medicine, “Sapienza” University of Rome, Rome, Italy; 2 Department of Radiological, Oncological and Pathological Sciences, “Sapienza” University of Rome, Rome, Italy; 3 Unit of Radiodiagnostics II, University Hospital “Policlinico Vittorio Emanuele”, Catania, Italy; 4 Department of Surgical Sciences, Radiology Unit, University of Turin, Turin, Italy

**Keywords:** Restrictive cardiomyopathies, Infiltrative cardiomyopathies, Cardiovascular magnetic resonance, Cardiac imaging

## Abstract

The restrictive cardiomyopathies constitute a heterogeneous group of myocardial diseases with a different pathogenesis and overlapping clinical presentations. Diagnosing them frequently poses a challenge. Echocardiography, electrocardiograms and laboratory tests may show non-specific changes. In this context, cardiac magnetic resonance (CMR) may play a crucial role in defining the diagnosis and guiding treatments, by offering a robust myocardial characterization based on the inherent magnetic properties of abnormal tissues, thus limiting the use of endomyocardial biopsy. In this review article, we explore the role of CMR in the assessment of a wide range of myocardial diseases causing restrictive patterns, from iron overload to cardiac amyloidosis, endomyocardial fibrosis or radiation-induced heart disease. Here, we emphasize the incremental value of novel relaxometric techniques such as T1 and T2 mapping, which may recognize different storage diseases based on the intrinsic magnetic properties of the accumulating metabolites, with or without the use of gadolinium-based contrast agents. We illustrate the importance of these CMR techniques and their great support when contrast media administration is contraindicated. Finally, we describe the useful role of cardiac computed tomography for diagnosis and management of restrictive cardiomyopathies when CMR is contraindicated.

## Introduction

Restrictive cardiomyopathy (RCM) is a myocardial disorder that is usually caused by increased myocardial stiffness which results in impaired ventricular filling. Until later stages of the disease, biventricular chamber size and systolic function are usually normal or almost normal. RCM include primary or idiopathic (a rare familial or sporadic genetic condition associated with the accumulation of desmin and collagen type III) and secondary forms, which include infiltrative, non-infiltrative and storage disorders.

RCMs are generally caused by processes causing abnormal deposition of proteins, glycogen and iron within the myocardium, leading to ventricular stiffness with diastolic dysfunction.

The most common classification system [1] in adults divides restrictive cardiomyopathies on the basis of etiology in:

1. Non-infiltrative: idiopathic RCM, scleroderma, pseudoxanthoma elasticum;
2. Infiltrative: amyloidosis, sarcoidosis, Gaucher’s, Hurler’s;
3. Storage diseases: Anderson–Fabry disease (AFD), glycogen storage, hemochromatosis and iron overload
4. Endomyocardial: endomyocardial fibrosis (EMF), radiation-induced, drugs, carcinoid, metastatic tumor.

The most common initial clinical manifestations are exertional dyspnea, exercise intolerance due to inability of the ventricular filling, fatigue and lower extremity edema, whereas heart failure symptoms occur only in advanced stage; atrial enlargement can lead to arrhythmias and concomitant thromboembolic complications are not uncommon [1].

Transthoracic echocardiography (TTE) is the first-line examination. The most common TEE findings are its normal right and left systolic function until advanced stage (normal ejection fraction), normal or reduced left ventricular (LV) volume and bi-atrial enlargement, with abnormal diastolic compliance characterized by increased early diastolic filling velocity due to elevated left atrial pressure.

Wall thickness is generally normal, except for infiltrative and storage processes in which case it is typically increased.

Cardiovascular magnetic resonance (CMR) can combine the morphologic and functional evaluation with an accurate characterization of the myocardial changes on the basis of the intrinsic magnetic properties of different tissues (T1, T2 and T2* values). Moreover, the use of gadolinium-based contrast agents (GBCA) may improve the evaluation of myocardial damage based on late gadolinium enhancement (LGE) technique and post-contrast T1 mapping sequences, which permit to calculate extracellular volume (ECV) [2]. The LGE phenomenon is due to the accumulation of GBCA in the extracellular space that can be increased in case of fibrosis, deposition of pathologic proteins or acute myocardial damage. Cardiac computed tomography (CCT) could be a valid alternative for diagnosis and management of infiltrative cardiomyopathies when CMR is contraindicated.

In this review, we illustrate the importance of these CMR techniques and their great support when contrast media administration is contraindicated. Finally, we describe the emergent role of CCT.

## CMR approach to restrictive cardiomyopathies

In case of suspected RCM, CMR protocol should encompass cine steady-state free precession (SSFP) electrocardiography-gated sequences to assess atrial and ventricular volumes, wall motion anomalies and morphologic abnormalities such as atrial enlargement or LV wall thickening [3].

**Fig. 1:**
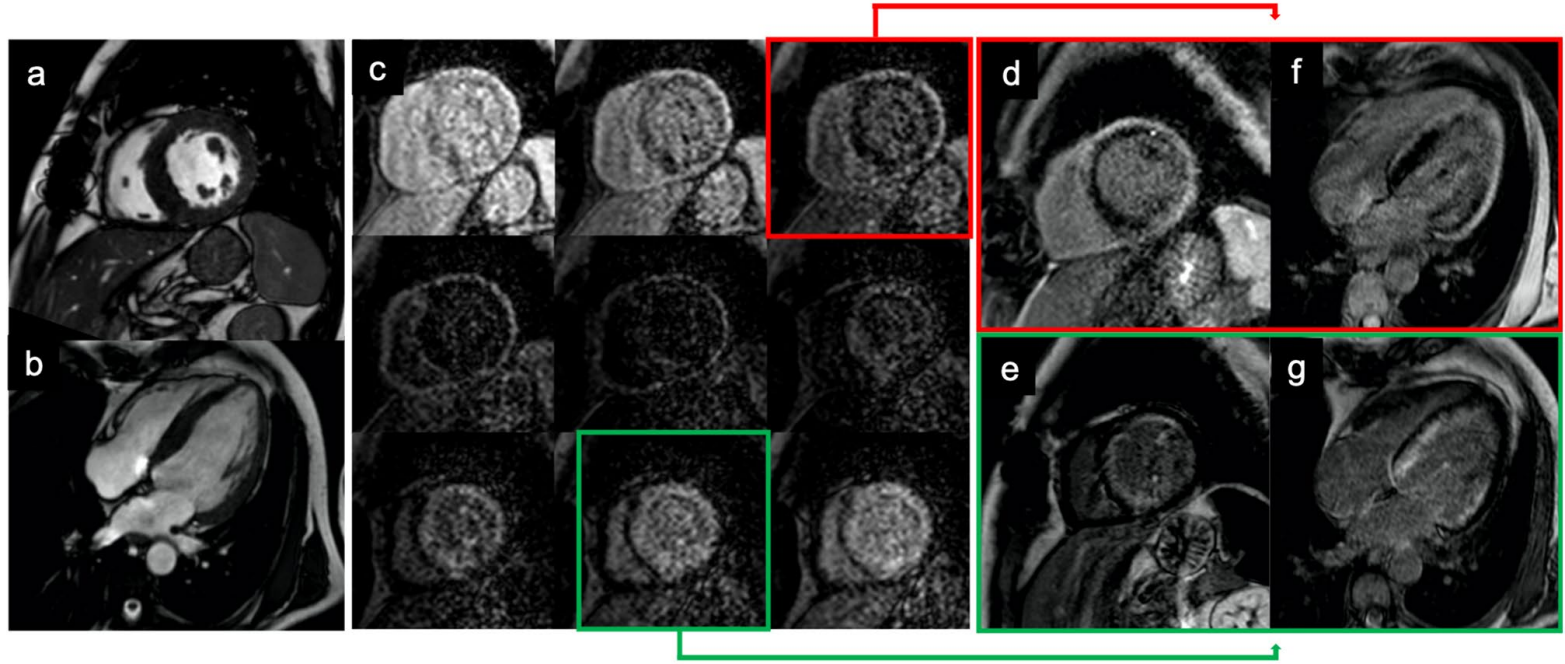
57-year-old patient with multiple myeloma with known bone lesion associated with light chain proteinuria and bilateral carpal tunnel syndrome. Cine-SSFP sequences (**a** short axis view; **b** 4-chamber view) showed a thickening of the left ventricular myocardium wall (19 mm in the septum) with global and moderate hypokinesia (left ventricular ejection fraction 46%). In panel **c** are reported some of the images of the lock–locker sequences of the TI scout. Inversion recovery turbo field echo sequences (**d** and **e** short axis view; **f** and **g** 4-chamber view) with a wrong myocardium null time (red box and arrow) and the one with the correct null time (green box and arrow); these last showed a diffuse areas of circumferential subendocardial pattern enhancement. The final diagnosis was light chain (AL) cardiac amyloidosis

LGE imaging is mandatory for differential diagnosis between forms of cardiomyopathies, because enhancement patterns may address distinct conditions. In traditional LGE imaging, the inversion time (IT) to null the signal of healthy myocardium is manually chosen by the operator using a specific sequence (Look Looker, for instance). The choice of the optimal IT is crucial to obtain maximum contrast between pathologic and normal cardiac tissue. This operator-dependent choice might be challenging in case of massive infiltration of the heart, for example in case of amyloidosis, and may result in erroneous choices resulting in non-diagnostic examinations (Fig. 1) [4]. The introduction of phase-sensitive inversion recovery (PSIR) sequences by all vendors, a LGE reconstruction technique less sensitive to operator choice of IT, has made it easier to obtain accurate LGE images and to determine the extent of cardiac involvement [5].

As they are currently available by all vendors, T1 and T2 mapping techniques should be used to characterize in a quantitative and reproducible way the tissue signal alterations in ICM and RCM [4, 5]. Native T1 (nT1) and post-contrast T1 maps are used to calculate ECV, a quantitative marker of myocardial fibrosis [6]. Conventional T2-weighted imaging and T2 mapping enable the detection of myocardial edema.

Finally, in case of suspected iron overload cardiomyopathy (IOC)-specific gradient echo sequences designed to measure T2* relaxation time should be incorporated into the CMR protocol in order to detect the excessive myocardial iron deposition (see specific section).

Main CMR features of RCM are summarized in Table 1.

**Table 1:** Main CMR features of restrictive cardiomyopathies

Disease	Wall thickness	Hypertrophy pattern	RV involvement	EDV	EF	LGE (pattern)	nT1	ECV	T2map	Atrial enlargement
Amyloidosis	↑↑	Asymmetrical (ATTR) Symmetrical (AL)	+	↑/~	~/↓	+(diffuse subendocradial/transmural)	↑↑	↑↑	↑/~	+
Fabry disease	↑↑ (male) ↑ (female)	Symmetrical	+	↓/~	~/↑	+(subendocardial inferolateral wall)	↓↓	↓/~/↑	↑/~	+/−
Iron overload	↑/~	Symmetrical	+	↑/~	~/↓	Not frequent (diffuse)	↓	↓/~/↑	↓	+/−
Radiation heart disease	~	No	+	↓/~	↓	+ (band-like, not specific)	↑	↑	~	+
Endomyocardial fibrosis	~	No	++	↓	↓	+(diffuse subendocardial)	~	~	↑/~	+

*RV* Right ventricle, *EDV* end-diastolic volume*; EF* ejection fraction*; LGE* late gadolinium enhancement, nT1 native T1, ECV extracellular volume, T2map T2 mapping. ↓/~/↑ increase/within normal range/reduced, ± :present/absent

## Amyloidosis

Amyloidosis is a group of diseases caused by protein misfolding resulting in aggregation and deposition of amyloid fibrils [7]. Cardiac amyloidosis (CA) occurs when the deposition of amyloid involves the extracellular space of the heart leading to organ dysfunction and adverse events. CA was thought to be a rare disease, but is currently considered an underdiagnosed condition [8].

More than 30 proteins are known to cause amyloidosis, and two types are predominantly responsible for cardiac involvement: immunoglobulin light chain amyloid (AL) and transthyretin amyloid (ATTR). ATTR amyloidosis is divided into a hereditary form associated with mutations of TTR protein (ATTRm), and a more common non-hereditary wild-type form (ATTRwt), a late-onset disease affecting predominantly men. ATTRwt almost exclusively affects the heart, while ATTRm has a wide range of presentations [7].

AL amyloidosis, the most frequent form of systemic amyloidosis, has an estimated prevalence of 8–12 cases per million person-years, and cardiac involvement occurs in 50–75% of cases [9]. CA is characterized by remodeling of the myocardium extracellular matrix, expansion of ECV, edema, reduction in capillary density, modifications in cardiomyocyte volume and even macroscopic changes in cardiac structure and function with the increase in LV mass and wall thickness resulting in diastolic dysfunction [5].

The reference standard for the diagnosis of CA is endomyocardial biopsy, an invasive procedure not widely available. In addition, the biopsy sample may not be representative of the infiltration status of the whole myocardium. Imaging offers a noninvasive alternative to evaluate the whole heart [4].

As CA is an important predictor of poor outcome, early diagnosis is crucial in both AL and ATTR amyloidosis. Imaging may guide the selection and the dosage of the chemotherapy to minimize treatment-related exacerbation of heart failure. In particular, CMR seems a promising tool to track different disease mechanisms (such as edema, infiltration and cardiomyocyte response) and to evaluate cardiac involvement progression or regression during the course of the therapy [10]. The identification of CA in ATTR patients permits to start treatment with targeted anti amyloid therapies.

CMR is a useful tool to differentiate the diagnosis between CA and other conditions (e.g., hypertrophic cardiomyopathy, hypertensive cardiopathy, AFD) [11]. CA was historically believed to be characterized by concentric symmetrical hypertrophy of the LV. However, CMR studies revealed that the most common phenotype of ATTR patients is asymmetrical LV hypertrophy (79% of cases). Asymmetrical septal hypertrophy is divided in two morphological subtypes: sigmoid septum (in 55% of ATTR patients) and reverse septal contour (in 24% of ATTR cases). No differences in morphological phenotype could be identified between the ATTRwt and ATTRm patients [12]. Symmetrical and concentric LV hypertrophy is present in only 18% of ATTR cases, while it is present in 68% of AL patients [12].

The evaluation of ventricular morphology and function in CA should be performed with cine images obtained using SSFP sequences acquired in long axis and short axis planes covering the LV.

LGE imaging with inversion recovery sequences is a fundamental technique to diagnose CA [11]. LGE imaging requires to set a proper inversion time to null the signal of healthy myocardium. This can be challenging in CA because of an accelerated clearance of gadolinium occurring when it encounters amyloid fibrils, or in case of diffuse infiltration, resulting in the difficulty of nulling myocardial signal before blood pool (Fig. 1). Such occurrences are highly suggestive for CA. The introduction of PSIR, an LGE technique less sensitive to operator choice of null point, has made LGE easier to perform on CA patients [13]. Three LGE patterns have been described: absent, diffuse subendocardial and transmural [13]. These patterns are correlated with the degree of infiltration of the LV and provide prognostic information, since a greater burden of infiltration is related to poorer prognosis [12].

**Fig. 2:**
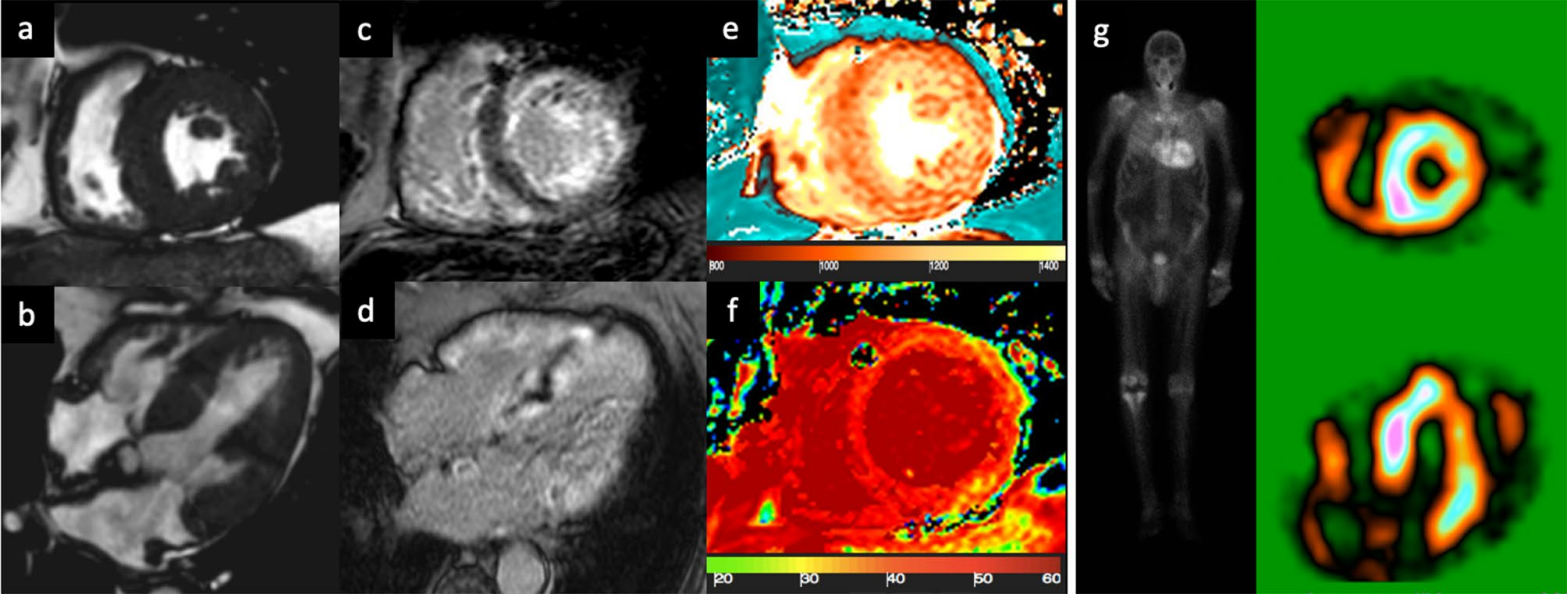
83-year-old male patient with known CAD and the presence of dyspnoea. Cine-SSFP sequences (**a** short axis view; **b** 4-chamber view), which show a thickening of both the left ventricular myocardium (18 mm in the septum) and the right ventricle, but also of the atrial walls with global and severe hypokinesia (left ventricular ejection fraction 26%). Inversion recovery turbo field echo sequences (**c** short axis view; **d** 4-chamber view) for late gadolinium enhancement (LGE) analysis; there are diffuse areas of circumferential subendocardial pattern enhancement even with transmural extension in the basal segment. There is also LGE within the right ventricle and both atrial walls. The quantitative evaluation of global left ventricular myocardium native T1 (**e** short axis view) and ECV (**f** short axis view) resulted in 1110 ms (v.n. 1000 ms) and 55% (v.n. 20–30%), respectively. Overall, the presence and the pattern of LGE with a transmural pattern in both ventricle and atrial walls were suspicious of transthyretin (ATTR) amyloidosis. The patient was then scanned with 99mTc-DPD (image **g**), where the abnormal and diffuse presence of the osteotropic indicator is observed in the left and right ventricle with a Perugini score = 3. The final diagnosis was ATTR amyloidosis

T1 mapping demonstrates an increased nT1 time in CA (Fig. 2) and is related to markers of systolic and diastolic dysfunction [14]. nT1 has been proposed as a quantitative technique to diagnose CA in patients with kidney failure and contraindication for GBCA [14]. Post-contrast T1 mapping and ECV estimation demonstrate markedly elevated ECV values in CA that are related to the prognosis [15]. ECV also provides insight into myocardial response to fibril deposition: Total myocyte cell volume, calculated from ECV and indexed LV myocardial volume, is higher in ATTR than AL patients, suggesting possible compensatory myocyte hypertrophy that might be protective [16]. ECV is a more robust prognosis marker compared with nT1 or post-contrast T1 value [17]. T1 mapping techniques seem to offer a more reproducible quantification of the infiltration burden compared with LGE [4]. T2 mapping in CA demonstrates increased T2 time in amyloidosis patients, but seems to be more variable than T1 mapping [18].

## Cardiac sarcoidosis

The development of caseating granulomas and tissue scarring represent the typical features of sarcoidosis, a systemic inflammatory disease that involves multiple organs and apparatus with an US annual prevalence of 10.9−35.5/100.000 in African-Americans and a cardiac involvement in 25% of subclinical patients [19].

**Fig. 3:**
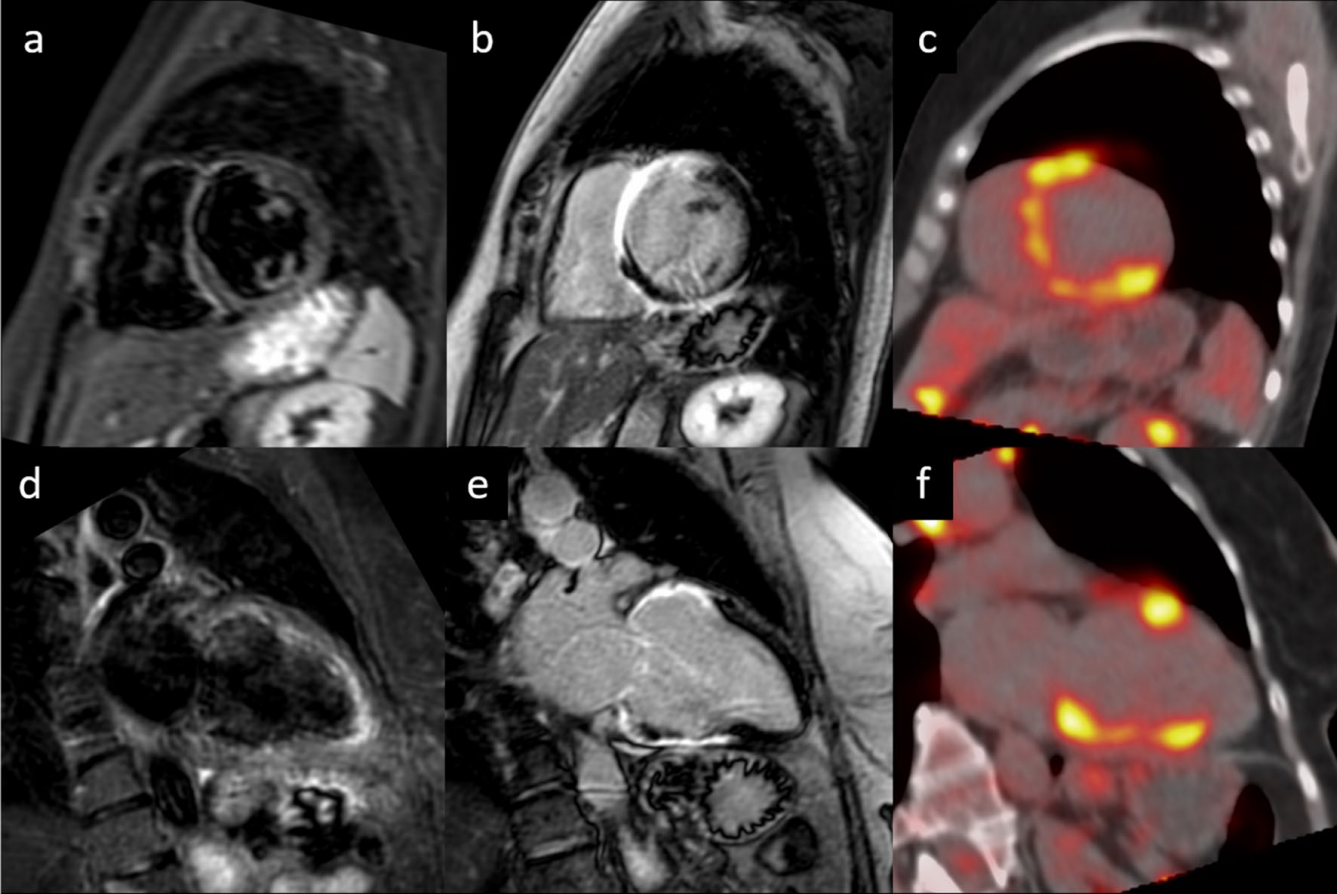
57-year-old female with frequent syncopal episodes and ventricular tachycardia, LV dilation and severe reduction in EF at TTE, with no obstruction of coronary arteries at coronary angiography. CMR revealed no edema on T2w-STIR images (**a** short axis view and **d** LV long axis view) and extensive areas of late gadolinium enhancement at IR-TFE images (**b** short axis view and **e** LV long axis view), with a non-ischemic pattern of distribution. The FDG-PET (**c** short axis view, **f** long axis view) confirmed the diagnosis of sarcoidosis with the identification of areas of FDG uptake (i.e., active inflammation) within the myocardium and in the mediastinal lymph nodes. *LV* left ventricle, *EF* ejection fraction; *TTE* transthoracic echocardium

A wide range of manifestations characterizes cardiac sarcoidosis (CS), the most common including complete heart block and right bundle branch block, ventricular arrhythmias and sudden cardiac death [20]. Diagnosis is challenging as ECG abnormalities are shown in just 3.2–8.6% of patients with clinically silent CS [20], and diagnostic criteria include different imaging modalities and histological confirmation [19]. In this setting, CMR represents a useful tool to better characterize myocardial tissue, with a reported negative predictive value of 100%, sensitivity of 100%, specificity of 78%, and diagnostic accuracy of 83% [20]. LGE (Fig. 3) is considered the most useful parameter with a typical mid-myocardial or subepicardial patchy distribution (in some cases it could be subendocardial), revealing areas of myocardial scarring and fibrosis, especially in the chronic phases of CS [21]. A patchy hyperintensity in T2-weighted sequences can be found in patients with active myocardial inflammation [20]. Several studies also showed a significant increase in native T1, T2 mapping and ECV values in patients with biopsy-proven extra-cardiac sarcoidosis as compared with healthy controls [19]. Mapping techniques improve the accuracy of CS diagnostic criteria and represent a helpful strategy to evaluate patients’ response to treatment, since mapping values seem to recover after immunosuppressive therapy in active CS [20]. Although CMR can investigate several aspects of cardiac involvement in sarcoidosis, the diagnosis remains challenging as its different features are often superimposable to that of acute or chronic myocarditis or to myocardial infarction. The novel hybrid imaging techniques like PET-CMR seem promising in the identification of active granulomatous lesions with high sensitivity [22].

## Lysosomal storage disease

The lysosomal storage disorders (LSD) and in particular the glycosphingolipidoses (Gaucher, Niemann–Pick and AFD), glycogen storage diseases (Pompe, Danon disease and PRKAG2 deficiency) and mucopolysaccharidosis frequently involve cardiac structures, causing infiltrative cardiomyopathies [23]. In the most common LSDs, cardiac involvement is characterized by ventricular wall hypertrophy, contractile impairment, arrhythmias, conduction abnormalities and progression to heart failure [24].

AFD is an X-linked disease (estimated incidence of 1/40.000–1/117.000 males) [24], caused by a mutation in the α-galactosidase gene, which leads to the multisystemic lysosomal accumulation of glycosphingolipids. AFD cardiomyopathy is a major determinant in patient’s survival and occurs in both classical phenotype (complete absence of enzyme’s activity) and variants (with very low residual α-galactosidase activity), which include the isolated form (“cardiac variant”) mimicking a sarcomeric hypertrophic cardiomyopathy.

AFD often appears as concentric LV hypertrophy with a variable degree of myocardial wall thickening and papillary muscles prominence, which lately (?) may lead to arrhythmias, myocardial ischemia or heart failure [1]. AFD usually becomes clinically apparent in the third decade of life in males, whereas in females it may be silent until much later in life [24]. While AFD diagnosis is often delayed, the prognosis is highly influenced by the timely start of enzymatic replacement therapy (ERT), as ERT inhibits the development and progression of myocardial damage [25]

On TTE, AFD phenotype overlaps many other hypertrophic conditions; therefore, the diagnosis may be challenging when not supported by specific ECG signs, positive familiar history or other manifestations of AFD. CMR has rapidly gained a crucial role in the diagnostics, as it combines the assessment of cardiac function and the characterization of tissue abnormalities [1].

**Fig. 4:**
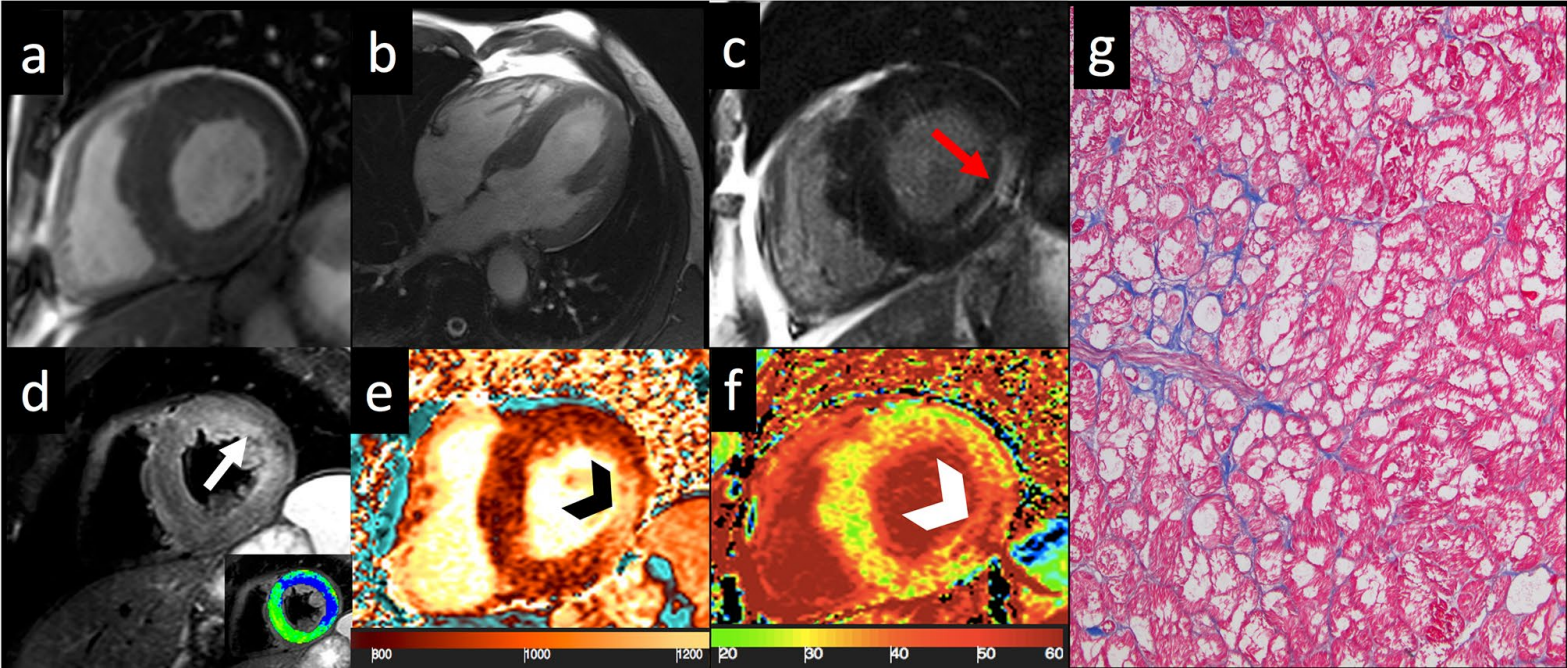
Anderson–Fabry disease—Cine-SSFP in short axis (**a**) and four-chamber (**b**) views acquired on end-diastolic phase demonstrate an asymmetrical hypertrophy with predominant involvement of septum (IVS maximal thickness: 20 mm). On LGE image (**c**), an area of mid-myocardial enhancement is detected in the LV inferolateral wall (red arrow). STIR T2-weighted image (**d**) shows an area of myocardial edema located in LV antero-lateral wall, with a subendocardial distribution pattern (white arrow), confirmed by the blue area (T2 ratio > 2) in the panel at the bottom. The analysis of nT1 (**e**) map demonstrates severe reduction in global nT1 (reddish brown color, nT1: 877 ± 23 ms, normal value for our scanner 970–1020 ms) except for the focus of increased nT1 at inferolateral wall (nT1: 1116 ms, black arrowhead) matching the area of increase in ECV (white arrowhead, ECV: 48%) on relative map (**f**, global ECV: 27%). Hematoxylin and eosin histology (**g**, ×200) shows cardiomyocytes hypertrophy, caused by large cytoplasmic and perinuclear vacuoles, containing myelin bodies. *Cine-SSFP* steady-state free precession images; *IVS* interventricular septum; *LGE* late gadolinium enhancement; *STIR* short tau inversion recovery; *LV* left ventricle; *nT1* native T1 map; *ECV* extracellular volume fraction

LGE has been found in up to 50% of AFD patients [26]; usually at the LV inferolateral wall with subendocardial involvement, which represents the typical hallmark on CMR (Fig. 4) [26], this typical LGE distribution is useful for differentiating diagnosis in the spectrum of LV symmetrical hypertrophy [27]. LGE has been explained as myocardial fibrosis due to the focal imbalance between the increase in collagen synthesis and decrease in metalloproteinases, caused by glycosphingolipids [28]. LGE together with the maximal wall thickness and cardiac mass represented the best predictor of cardiac events [29], even if in women LGE frequently occurs before LV hypertrophy development [30].

As validated in various studies [31–34], myocardial nT1 values are globally decreased, as a consequence of the myocardial accumulation of glycosphingolipids [31], and may discriminate from other infiltrative cardiomyopathies that, apart from iron overload, are usually associated with normal or incremented T1 values [32, 33].

In a study performed on 123 patients, nT1 distinguished AFD from hypertrophic cardiomyopathy (HCM) and healthy controls (sensitivity 88% and 88%, specificity 92% and 86%, respectively) using a cutoff value of 940 ms on a 1.5 T scanner and modified lock–locker inversion (MOLLI) recovery sequence, whereas a better diagnostic performance is obtained with 3.0T scanner (sensitivity 97%, specificity 93%, threshold of 1220 ms) [35]. Myocardial nT1 lowering has also been observed in 41–59% of AFD patients with no LV hypertrophy [32, 36], thus appearing an early marker of disease progression and predictor of clinical worsening at a 12-month follow-up [36]. Myocardial ECV is generally preserved in AFD patients (with lower values in males compared to females [33]).

In a recent study, an increase in T2-weighted signal was found in 24/78 AFD patients reflecting myocardial edema, whereas myocardial inflammation was confirmed by histology in 44/78 patients [34]. Other authors found an elongation of T2 relaxation time (global or localized to segments with LGE) as compared to HCM and controls [37], associated with chronic troponin elevation, suggesting the presence of an underlying chronic inflammatory cardiomyopathy [34].

Although systolic function assessed by ejection fraction is generally normal in AFD patients, different studies reported myocardial strain reduction assessed with feature tracking (FT) technique. In particular, AFD patients show impairment in global longitudinal strain (GLS) correlated with myocardial damage degree (presence of LGE and cardiac biomarkers) and nT1 values, even at pre-hypertrophic stage [38]. Furthermore, AFD is characterized by reduction in global circumferential strain (GCS) and in GCS gradient from the LV base to the apex [39].

Finally, the role of CMR to assess the response to ERT is under investigation. It has been found that ERT induces a decrease in myocardial T2 values, and in LV mass and wall thickness of patients with little or no LGE at baseline [25]. ERT also causes a slight increase in T1 mapping values, especially in patients at earlier stages of the disease, while its effects seem less effective in more advanced disease [25].

## Iron overload

Iron overload or hemochromatosis indicates accumulation of iron in the body due to genetic metabolic disorders with increased intestinal iron absorption (primary form) or repeated blood transfusions (secondary form) [40]. When involved, the heart can develop a secondary cardiomyopathy, the IOC, which is the leading cause of death and is characterized by cardiac dysfunction, initially diastolic and then systolic, secondary to increased deposition of iron in the myocardium [40].

Myocardial iron overload is a progressive process depending on the increasing levels of serum iron, regulated through transferring mediated uptake mechanisms. In the cardiomyocytes, iron deposition initially occurs in the perinuclear lysosomes, but when the overload exceeds, iron can accumulate throughout the sarcoplasm [40, 41].

Myocardial iron deposition initially begins within the epicardium and then extends toward the endocardium, which helps explain the preservation of systolic function until very late in the disease. The excess of iron may be removed with chelation therapy, even though the reversibility of myocardial damage is reduced in the more advanced stages of the disease [41].

Clinical presentation of IOC varies from the total absence of any symptoms or only exertional dyspnea at early phase to heart failure symptoms, when the damage severely affects LV systolic function and determines dilated cardiomyopathy [41]. Right heart failure can also be present. Iron deposition can also occur in the pericardium, and if extensive enough, it results in clinical signs and symptoms [42].

TTE is commonly used to screen the patients and for clinical follow-up. It is able to detect abnormalities in terms of wall thickness or myocardial contraction, but it may not directly visualize tissue iron deposition [41]. At TTE, LV usually shows normal wall, biventricular dilatation and progressive evidence of a restrictive pattern.

**Fig. 5:**
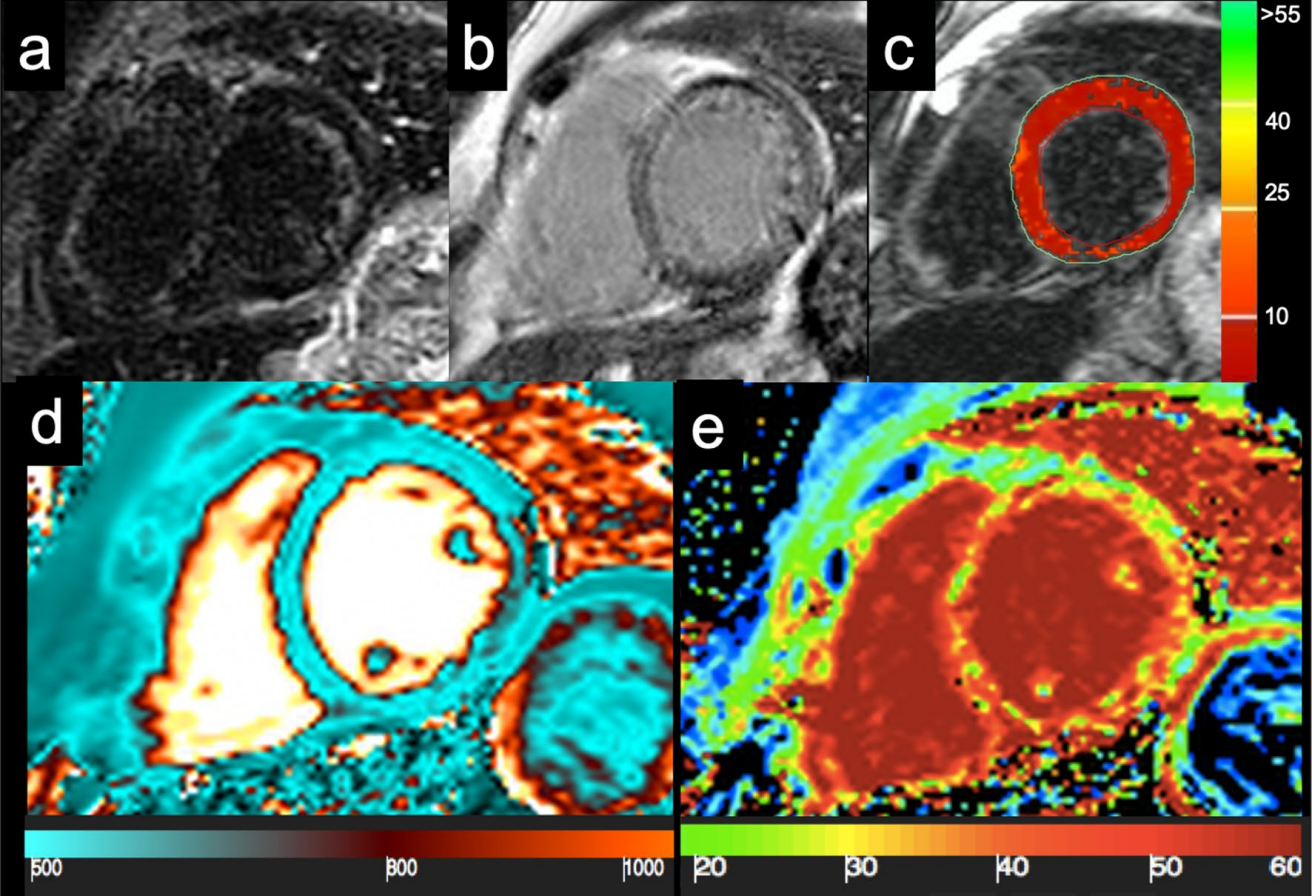
Cardiac iron overload—A 42-year-old woman with Cooley’s disease and moderate reduction in ventricular function (EF: 42%) show a global myocardial hypointensity on STIR image (**a**) and a diffuse inhomogeneous abnormal signal on LGE imaging (**b**), with no evidence of clear focal areas of enhancement. Analysis of T2* map (**c**), generated by traditional multiecho gradient echo T2-weighted sequence, shows a diffuse and marked reduction in the global myocardial T2* relaxation time (T2* = 0–1.5 ms, normal value > 20 ms). nT1 map (**d**) shows a significant reduction in global nT1 value (nT1 ≈ 535 ms, normal value 970–1020 ms), affected by susceptibility effect of intramyocardial iron accumulation. ECV map **e** reveals diffuse fibrosis (ECV ≈ 38–40%). *STIR* short tau inversion recovery; *LGE* late gadolinium enhancement; *nT1* native T1 value; *ECV* extracellular volume fraction

CMR has emerged as the best noninvasive method to quantitatively assess the myocardial iron load [40, 42]. In non-iron overloaded hearts, the signal is homogeneous and relaxation time lasts for a longer duration. In IOC, the paramagnetic effect of iron produces changes in MR signal intensity and shortens T1 and T2 relaxation times (Fig. 5).

In particular, iron deposits create a rapid signal loss with increasing echo time that can be better assessed as a reduction in T2* time, calculated with an exponential function using a multiecho gradient echo sequence [43]. T2* measured in a full-thickness region of interest within the interventricular septum is considered highly representative of global myocardial iron [43].

A value of 20 ms is considered the best performing threshold to define myocardial siderosis at 1.5T scanner [41]. Myocardial T2* values > 20 ms measured on 1.5T scanner (12 ms on 3.0T scanner), corresponding to lack of iron overload or benign iron load, are associated with normal cardiac function with a high negative predictive value.

T2* value ranging from 10 ms to 20 ms at 1.5T (from 5.5 ms to 12 ms at 3.0T) is highly indicative of moderate myocardial siderosis and it is correlated with LV ejection fraction [44], and T2* values < 10 ms (< 5.5 ms at 3.0 T) are indicative of severe iron overload, associated with an increased risk of the development of heart failure or arrhythmias [41, 44].

T2* relaxation time is not correlated with serum ferritin levels, although there is a strong correlation with the quantified amount of iron deposition from myocardial biopsy [41]. T2* imaging has also emerged as the best quantitative parameter to guide and assess response to chelation therapy [45] and to monitor disease progression, and is currently the only parametric mapping technique recommended in disease-specific clinical guidelines [46].

Although the T2* technique remains the reference technique for clinical assessment of iron overload, great advantages are offered by the novel T1 and T2 mapping sequences, since myocardial values are reduced in both sequences [46].

In addition, in patients with only mild increases in cardiac iron, nT1 showed a superior reproducibility as compared to T2* measurements (about 2.5–7 fold T2*) [47].

LGE was also detected in the 15,6–19% of patients with Thalassemia Major and the extent of myocardial fibrosis was comparable in patients that developed heart failure and patients who did not [48].

Finally, ECV may be increased in patients with cardiac iron overload, as it reflects diffuse interstitial myocardial fibrosis that occurs in more advanced phases of the disease [46].

## Endomyocardial fibrosis

EMF is characterized by deposition of fibrous tissue in the endocardium leading to restrictive pattern, with the reduction in ventricular volumes and increase in atrial volumes, normal wall thickness or apical obliteration due to fibrous endocardial thickening [49].

EMF was initially described in tropical countries, in young adults with a bimodal distribution peaking at 10 and 30 years of age, mainly in rural and poor populations [49].

Although the pathogenesis is unclear, malnutrition, parasitic infestation (malaria, schistosoma, filariasis), genetic factors have been proposed as potential causes triggering inflammation and immunomodulation. Hypereosinophilia, infection disease and autoimmunity may act as a profibrotic role by promoting the synthesis of collagen by fibroblasts, with progressive endomyocardial damage and scarring.

EMF usually starts with active diffuse inflammation with endothelial damage, myocardial edema, eosinophilic infiltration and subendocardial necrosis and vasculitis, frequently associated with pericardial effusion and thrombi adherent to endocardial surfaces. When the inflammatory activity declines, it evolves in a progressive interstitial fibrosis and myocyte hypertrophy (chronic phase) causing RCM, whose phenotype is characterized by biventricular volume reduction and atrial dilatation, and subsequent isolated right-sided heart involvement with apical retraction.

Extensive EMF may cause restrictive patterns, apex obliteration (differential diagnoses includes apical HCM) and diastolic dysfunction.

CMR offers a comprehensive evaluation of ventricular function and morphology (including an excellent visualization of the ventricular apex), endocavitary thrombus detection and assessment of tissue abnormalities.

**Fig. 6:**
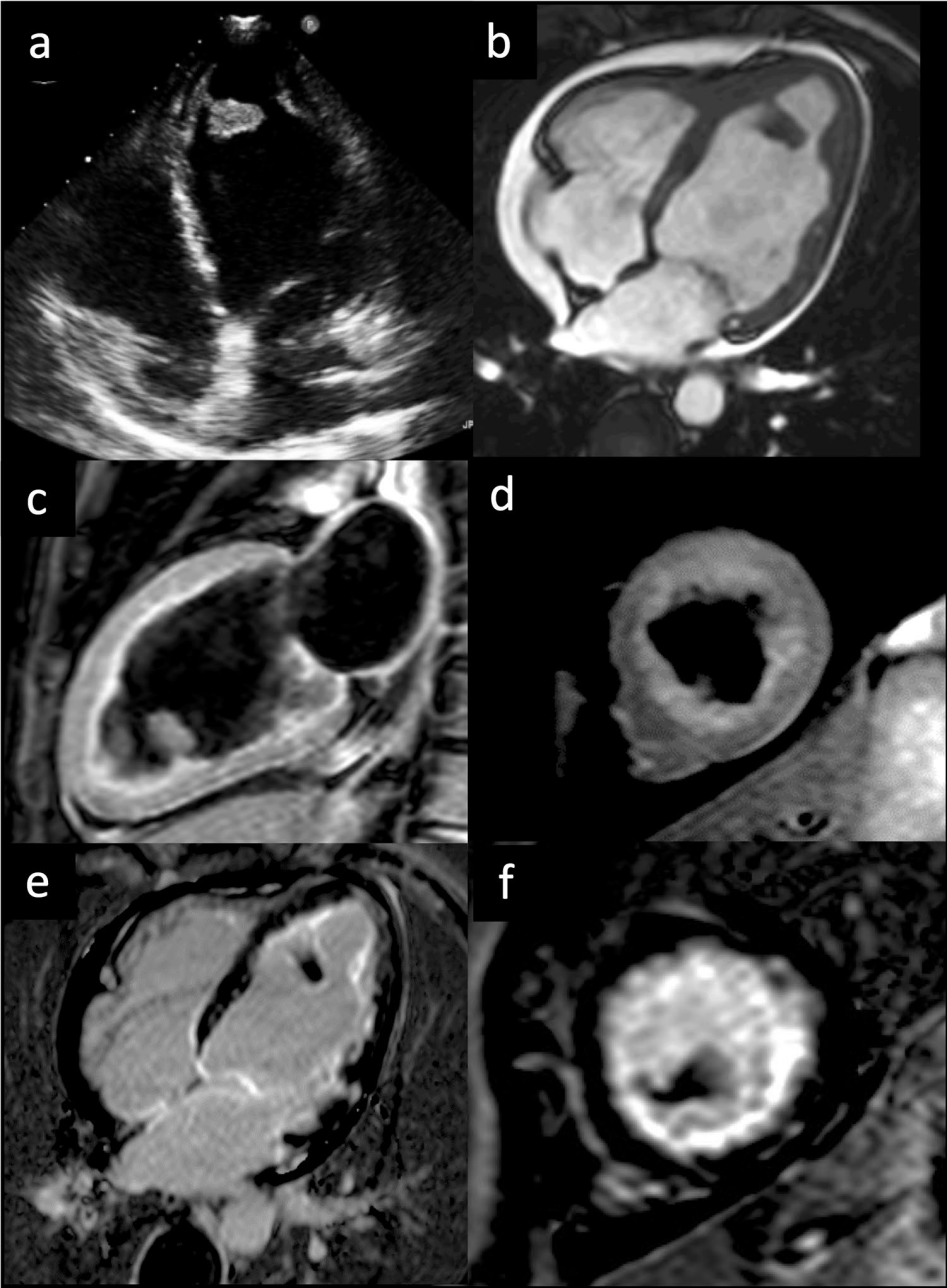
Endomyocardial fibrosis—40-year-old patient with hypereosinophilia. Transthoracic echocardiogram in 4-chamber projection demonstrates the presence of an apical thrombus (**a**). Cine-SSFP in 4-chamber view (**b**) confirms echocardiographic findings and evidence a reduction in EF (35%). STIR in 2-chamber plane (**c**) and short axis (**d**) shows no signs of edema. LGE images in 4 chambers (**e**) and short axis (**f**) demonstrate a diffuse subendocardial hyperintensity and a circumferential pericardial effusion

LGE typically involves the endocardial and subendocardial layers of both ventricles; with a non-coronary pattern, and the subvalvular apparatus and chord, LGE is typically a continuous hyperintense stria extending from the subvalvular region to the apex, where it is usually more prominent (Fig. 6) [50].

In this setting, CMR has a role not only for diagnosis and staging, but also in assessing response to treatment, in inhibiting inflammation and solving thrombosis, and in prognostic stratification [49].

## Radiation-induced heart disease

Radiation therapy is a valuable therapeutic option, which improves clinical outcome and reduces post-surgery recurrences in various thoracic malignancies (e.g., Hodgkin or non-Hodgkin lymphomas, breast or esophageal cancer). Among potential complications, cardiovascular diseases are among the most serious and for a long time were underestimated.

The term “radiation-induced heart disease” (RHD) depicts a complex entity that can manifest in a large number of conditions, such as accelerated atherosclerosis, valve disease, cardiomyopathies, conduction system abnormalities and pericarditis [51]. RHD is influenced by many factors (e.g., the dose, interval between irradiations, concomitant chemotherapy) and its incidence is ranging from 0.5 to 37% among patients treated for breast cancer and 49.5–54.6% for lymphoma, with an overall estimated prevalence of 10% [52]. Commonly, RHD patients remain asymptomatic for a long time, and only 10% manifest symptoms or signs decades after treatment [53].

A spectrum of different phenotypes are associated with RHD, including myocardial fibrosis with wall motion anomalies, LV hypertrophy, diastolic dysfunction with restrictive phenotype and congestive heart failure, and are quite common in patients who received more than 60 Gy or chemoradiotherapy [52].

In this clinical setting, the role of CMR for the assessment of RHD has not been fully established.

CMR appears promising in anticipating the RHD diagnosis by depicting the early myocardial tissue changes related to the radiation-induced damage before the occurrence of functional impairment, with consequent impact in patient management and treatment strategy [54].

Very few studies investigated RHD using CMR. Umezawa et al. [55] found LGE in 52% of 24 patients treated for esophageal cancer with a predominant mid-layer myocardial distribution, associated with hypokinesia of LGE + segments. Another study by Machann et al. [56] on 31 patients with history of mediastinal RHT for Hodgkin’s disease reported subendocardial or transmural LGE in 26% of patients, and perfusion defects, evaluated with stress CMR, in 61% of the patients.

The quantitative biventricular functional assessment offered by cine sequences is recommended in those with suboptimal TTE or discrepant results on 2016 ESC Position Paper on Cancer Treatments and Cardiovascular Toxicity [57]. Pericardial thickening is also frequent in those patients [58].

Novel T1 and T2 mapping techniques could open new perspectives in the early detection of diffuse myocardial inflammation and fibrosis following RHT. However, systematic studies are still lacking and preliminary experiences have been reported only as isolated case reports [59].

## Differential diagnosis between RCM and constrictive pericarditis

The distinction between constrictive pericarditis (CP) and RCMs could be challenging, as the two manifest with overlapping clinical presentations and restrictive flow patterns with diastolic dysfunction at TTE [60].

RCMs are associated with increased stiffness and reduced relaxation of ventricular walls, which alter the elastic properties or the extracellular matrix of the myocardial tissue.

Conversely, CP is typically a complication of chronic pericarditis or pericardiotomy and determines an encasement of cardiac chambers in a rigid pericardial sac, resulting in interventricular dependence and dissociation between intracardiac and intrathoracic pressures during respiration [61].

CMR provides useful information that helps to differentiate the diagnosis between CP and RCMs.

First of all, in CP “*black blood*” T1-weighted sequences and cine-SSFP can demonstrate a diffuse thickening of pericardial layers with calcifications (typically hypointense) [62], even if almost 20% of CP patients have normal pericardial thickness [61].

Pericardial effusion can be present in both CP and RCM (i.e., in amyloidosis), but in RCM it does not show septa or loculations, which are typical of CP [62]. Atrial enlargement is a characteristic feature of RCMs, together with ventricular wall hypertrophy [62].

Contrast enhancement of the pericardial layers has been reported in 48–73% of patients with CP and is supposed to be associated with neovascularization and chronic inflammation. Therefore, it could be considered a predictor of the reversibility of CP after treatment with anti-inflammatory agents. On the other hand, the presence of hyperintense pericardial signal on T2-short tau inversion recovery is a sign of active inflammation, and it has been reported in just 3% of patients with CP [63].

**Fig. 7:**
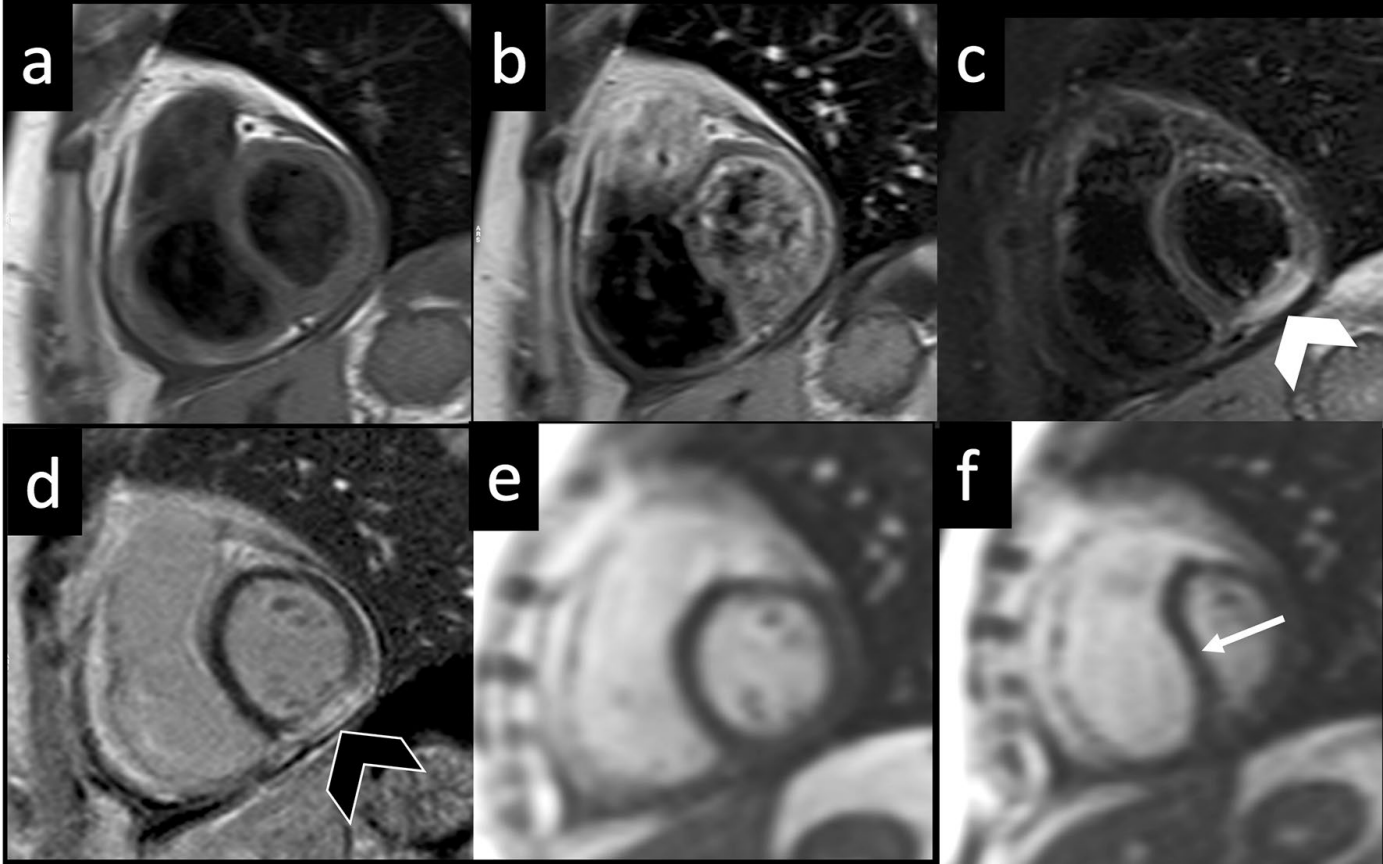
Constrictive pericarditis—TSE T1-weighted images acquired on short axis view before (**a**) and 3 min after gadolinium administration (**b**) demonstrate a diffuse thickening of pericardial layer with minimal effusion and slight enhancement of the visceral pericardial layer. On STIR T2-weighted image (**c**), a focal area on myocardial edema (white arrowhead) is found in the LV inferior wall corresponding to the area of pathological enhancement (black arrowhead) on LGE image (**d**) by demonstrating a condition of active myocarditis. On free-breathing real-time cine-SSFP acquired in end-expiration (**e**) and end-inspiration (**f**), a bouncing and leftward shifting of IVS is seen during inspiration (white arrow) due to the inversion of interventricular pressure ratio combined with inextensibility of the pericardial sac. *TSE* turbo spin echo; *STIR* short tau inversion recovery; *LGE* late gadolinium enhancement; *LV* left ventricle; *SSFP* steady-state free precession; *IVS* interventricular septum

Free-breathing real-time cine sequences are able to depict in CP interventricular septal flattening or bouncing during inspiration. This is related to the pathological ventricular coupling and seems to be more pronounced in the early ventricular filling (Fig. 7) [64], whereas in RCM septal shape does not show any respiratory-related changes [65].

According to Power et al., tagged cine-MRI enabled the assessment of adherences between visceral and pericardial layers of pericardium by identifying the absence of slippage, with a sensitivity and diagnostic accuracy of 100% [66].

## Multimodal approach: usefulness of cardiac CT

Cardiac computed tomography angiography (CCTA) has a role for disease characterization in patients with suspected RCM with contraindication to CMR (e.g., severe claustrophobia, non-MRI conditional pacemakers), as well as to exclude coronary artery disease.

Retrospective ECG-gated CCTA scan with multiphase reconstruction may assess ventricular wall thickness, shape and volumes, with excellent correlation to both TTE and CMR [67]. CCT can also identify thickening, calcification or enhancement of the pericardial layers or pericardial effusion, helping the diagnosis of pericardial diseases, which may go in differential diagnosis with restrictive cardiomyopathies [68].

LGE is the cornerstone of differential diagnosis of cardiomyopathies in CMR and can be demonstrated also with CCT [69]. The late iodine enhancement (LIE) can be imaged in CCTA because iodine contrast medium and GBCA have similar kinetics resulting in comparable washin and washout for both healthy and pathologic myocardium [70]. LIE imaging with CT can be obtained 5–15 min after contrast injection [71].

Although LIE can be demonstrated with single energy scanners [72], dual energy CT scanners with techniques of material decomposition may also provide iodine maps for an even more confident diagnosis [73].

ECV has been demonstrated to be a robust indicator of disease burden and a prognostic marker in CA and its results are altered in other ICM [4]. ECV can be derived from CCT examinations by combining non-contrast and contrast-enhanced acquisitions. ECV obtained from CT examinations seems to have a good correlation with ECV obtained from CMR [71]. Nevertheless, it is important to emphasize that the operator’s experience is crucial to identify LIE [74].

The advantages of CCT imaging consists in wide scanner availability, reduced costs and examination time, simultaneous evaluation of the coronary arteries and the possibility to perform in patients with CMR contraindications. Disadvantages are a less robust tissue characterization compared with CMR and the patient exposure to ionizing radiations and iodinated contrast medium.

## Conclusion

CMR is emerging as a robust and powerful noninvasive imaging modality for the diagnosis of various forms of ICMs and RCMs. The myocardial tissue characterization offered by CMR and strengthened by the novel T1 e T2 mapping technique, may identify those storage or infiltrative forms that are associated with characteristic alterations of the myocardial relaxometric properties or detect other restrictive conditions, where myocardium is subject to diffuse inflammation or fibrosis. When the clinical scenario is unclear, a CMR multiparametric approach may help to reach the correct diagnosis with a significant impact on clinical decision making.
